# “We must be mentally strong”: exploring barriers to mental health in correctional services

**DOI:** 10.3389/fpsyg.2024.1258944

**Published:** 2024-01-23

**Authors:** Ryan Coulling, Matthew S. Johnston, Rosemary Ricciardelli

**Affiliations:** ^1^Providence University College and Theological Seminary, Otterburne, MB, Canada; ^2^Memorial University of Newfoundland, St. John's, NL, Canada

**Keywords:** correctional workers, mental health, stigma, occupational hierarchy, organizational change

## Abstract

**Introduction:**

The inherent nature of work in correctional services can have negative effects on correctional worker mental health and well-being.

**Methods:**

The current study, a replication, analyzes survey data collected from provincial and territorial correctional workers staffed in six regions across Canada (*n* = 192). Specifically, participants were asked at the end of an extensive mental health and well-being survey an open-ended question requesting any additional feedback or information.

**Results:**

Four predominant themes were identified in the data: (1) stigma pertaining to a need to recognize mental health concerns within correctional services; (2) the idea that correctional services wear on the mind and body; (3) a need for better relationships with and support from correctional supervisors, upper management, and ministerial leadership; and (4) suggestions to improve correctional services to help the sector realize its full potential and maximize workplace health.

**Discussion:**

We discuss the implications of these findings, with an emphasis on finding ways to promote positive organizational and cultural change in correctional services.

## Introduction

Correctional work unavoidably has negative effects on correctional worker mental health and well-being (Boudoukha et al., 2011; Denof and Spinaris, 2016; James and Todak, 2018; Genest et al., 2021; Johnston et al., 2021, 2022b; McKendy et al., 2023; Ricciardelli et al., 2023b). Correctional workers, whether working in prisons, the community, or administrative roles, are at an increased risk to screen positive for mental health disorders. For example, Regehr et al. (2019) found higher rates of Posttraumatic Stress Disorder (PTSD), Major Depressive Disorder, and Generalized Anxiety Disorder among correctional workers than other professions and the general population. In their study of Canadian public safety personnel, which included correctional workers, researchers found 54.6% of correctional workers screened positive for one or more mental health disorders (Carleton et al., 2018, 2022; Ricciardelli et al., 2018). This finding is considerably higher than the 44.5% prevalence among all public safety personnel this same research reported, and also remarkably higher than findings among the general population in Canada, in which one in three people will be affected by a mental health disorder during their lifetime (Government of Canada, 2020) and 10.1% will be diagnosed with a mental health disorder (Statistics Canada, 2020).

The evidence detailing the presence of mental health disorders in correctional work continues to evolve (Ricciardelli et al., 2023a). For example, Ricciardelli et al. (2023a) found that recent divestment in rehabilitative programs and reductions in funding exacerbated prison conditions in Ontario, Canada, whereby correctional officers endured greater exposure to potentially psychologically traumatic events (PPTE), which can shape mental health outcomes, including the onset of mental health disorders. Earlier, Ricciardelli et al. (2019) detailed the prevalence of positive screens for mental health disorders among various types of federally-employed correctional workers that include Major Depressive Disorder, PTSD, and Generalized Anxiety Disorder. They found that 57% of correctional workers working in operations within prisons, 45% of correctional workers working in the community, and 56% of administrative workers in regional and national headquarters screened positive for at least one mental health disorder. Carleton et al. (2020) also found that 58.2% of provincial correctional workers working in Ontario screened positive for a mental health disorder, 11.5% had two positive screens, and 26.9% had three or more positive screens. From this same population of correctional workers, the researchers also found that 30.7% screened positive for PTSD, 37.0% screened positive for Major Depressive Disorder, 30.5% screened positive for Generalized Anxiety Disorder, 14.1% screened positive for Panic Disorder, and 6.7% screened positive for Alcohol Use Disorder.

In the current study, we extend the literature by confirming and building on previous findings about correctional worker mental health and wellness. The purpose of our research is to explore what considerations respondents (*n* = 192) felt were most important to emphasize at the end of an extensive mental health and well-being study. Themes identified in the responses include stigma pertaining to a need to recognize mental health concerns within correctional services; the idea that correctional work and services (operationally or organizationally) wear on the mind and body; a need for better relationships with and support from correctional supervisors, upper management, and ministerial leadership; and suggestions to improve correctional services to help the sector realize its full potential and maximize workplace health.

### Correctional workers, PPTE, and trauma

Much of correctional workers’ trying mental health experiences and status may be explained by the nature of their work, in both community and prison settings, including elements that emerge from their operational job duties or are a result of structural aspects of correctional services (Ricciardelli and Power, 2020; Norman and Ricciardelli, 2022; Ricciardelli et al., 2023a). Occupational stressors and PPTE occur from assaultive harms, routinized violence, verbal aggression directed at correctional workers (McKendy et al., 2021), and from witnessing and responding to harm, such as incarcerated people engaging in self-harm, suicide behaviors or completion, and violence between incarcerated people (Boudoukha et al., 2011; Viotti, 2016; Barry, 2017; Walker et al., 2017; Lerman et al., 2022). Given the complex and intensive nature of prison spaces (Levan, 2016), the vast majority of correctional workers experience PPTEs. In one study, 90.2% of correctional workers in Ontario were exposed to physical assault and 81.8% were exposed to sudden violent death (Ricciardelli et al., 2022). Further, these stressors and PPTEs are aggravated because correctional workers continue to work in the spaces where these events occurred.

PPTEs can also emerge from interactions between coworkers and managers. In their research of provincial and territorial correctional workers in Canada, McKendy et al. (2021) discovered how verbal assault, ranging from harassment to bullying to discrimination, and physical assault and victimization among correctional workers, remains prevalent. These researchers also uncovered how difficult managerial relations could aggravate the harm endured from incarcerated people, coworkers, and other managers because managers either failed to respond to the concerns of frontline staff, or responded problematically (i.e., victim blaming). These types of problematic relations can render some correctional workers feeling devalued and distrustful of management (McKendy and Ricciardelli, 2022). Other structural aspects of the job, including issues related to workload, labor shortage, shift work, contractual employment, wages, and benefits (Triplett et al., 1996; Keinan and Malach-Pines, 2007; Swenson et al., 2008; Morse et al., 2011; Brower, 2013; Ricciardelli et al., 2020), have also been found to worsen correctional worker stress and frustration, which are factors that may compound to degenerate correctional worker wellness and produce deleterious mental health outcomes.

### Distinguishing between operational and organizational factors

While there are myriad occupational factors that can lead to mental health concerns among correctional workers, following Duxbury et al. (2015), we catalog these into one of two classifications of stressors: operational and organizational. Operational stressors pertain to the stressors that arise from the job content (Norman and Ricciardelli, 2022; Ricciardelli and Carleton, 2022). These are the strains that are mostly unique to the profession as they emerge directly from the work itself. Being spat on by an incarcerated person, witnessing and responding to incarcerated people harming themselves or others, and the violence that occurs within correctional facilities are all examples of operational stressors. Organizational stressors, on the other hand, are stressors that emerge from job contexts (Norman and Ricciardelli, 2022; Ricciardelli and Carleton, 2022). Generated from the structural elements of an occupational organization, these are stressors that can arise from aspects that are common in many organizations. These might include perceived issues with management styles and worker support or issues pertaining to workload and wages (Duxbury et al., 2015).

The current study benefits from distinguishing between operational and organizational stressors in two ways. First, understanding the type of stressor allows our analysis to inform recommendations at the appropriate level of correctional services, as well as distinguish between the stressors that are easy or difficult to change, given the inevitably of stressors in correctional services or public safety professions more broadly. Second, it is important to acknowledge how each type of stressor may lead to different mental health concerns. Regehr et al. (2019), for example, found that physical violence and injury—operational stressors—were most strongly associated with PTSD, while low support and job satisfaction, and a lack of appreciation—organizational stressors—were more apt to lead to symptoms of depression and anxiety.

### Stigma and the desire for recognition of mental health needs

Despite the prevalence of mental health concerns among correctional workers that can be explained, to varying degrees, by operational and organizational factors within correctional services, mental health is still stigmatized in the profession, despite on-going suggestions for improvement and increased recognition of the mental health plights of correctional workers, in Canada especially (see Johnston et al., 2022b; Johnston and Ricciardelli, 2023). Johnston et al. (2021) have illustrated how stigma materializes within correctional services when mental health becomes framed as a personal problem to be resolved by the individual, rather than through a mutual partnership between the worker and organization. Even though it is well-established that the majority of correctional workers will encounter mental health challenges at some point in their career, the individualist responsibilization of mental health forms a culture of silence that intensifies correctional workers’ feelings that they need to hide their mental health concerns and suppress emotions and discussions related to mental health (Westaby et al., 2020).

Individualizing mental health further shapes treatment-seeking practices, or lack thereof. Johnston et al. (2021) recognize how some correctional workers believe they can neither share their awareness of mental health concerns, nor encourage solutions within the correctional culture, though Johnston et al. (2022b) documents how, more internally, many correctional workers have thought critically on this topic and have produced several reasonable avenues for positive change in correctional services. Yet before correctional workers can fully actualize these possible solutions for mental health, stressors, and exposure to PPTEs, stigma around mental health must be addressed. Research finds the first step to reducing stigma is for both frontline correctional workers and management to openly acknowledge and discuss as a team the mental health needs of correctional workers, the effects these underrecognized and underserved burdens have on correctional services, and the role the nature of the correctional work has on correctional workers’ mental health (Ricciardelli et al., 2020).

Further, a cultural environment where discussions about mental health are normalized and in which correctional workers can openly turn to peers and peer supports may help to reduce stigma (Johnston et al., 2022b, 2023). Relatedly, in their survey research of 118 healthcare professionals working for the Italian Health Authority, Paganin et al. (2023) found an indirect effect of mental health leadership on teamwork through interpersonal conflict and cooperation, suggesting leadership training and frameworks can positively impact teamwork among workers, particularly amidst crises such as the COVID-19 pandemic (see also Franke et al., 2014). Together, these findings point to the necessity of correctional services facilitating pro-social relational workplace dynamics and effective leadership practices.

## Methods

### Materials

We draw on data collected from the Correctional Worker Mental Health and Well-being Study, which comprised an extensive online survey with the overall objective of contextualizing the mental health, well-being, and other factors potentially impacting the health of correctional workers. The data were collected prior to and up until the declaration of the COVID-19 pandemic. Between early 2018 to early 2020, we administered the survey in the provinces of Manitoba, Saskatchewan, Nova Scotia, New Brunswick, Newfoundland and Labrador, and Yukon Territory.

### Procedure

The survey was anonymous, confidential, and voluntary. We received ethics approval from research ethics boards at the University of Regina (file #2017-098) and Memorial University of Newfoundland (file #20201330-EX). Agreeing to consent was solidified before anyone could access the survey. We informed participants to contact their Employee Assistance Program or alterative supports if they had thoughts of “being better off dead,” of harming themselves, or of suicide. We also developed protocols if participants required immediate help; protocols included a website listing crisis centers, calling 911, or visiting their nearest organization for emergency intervention.

Survey distribution, as well as recruitment, occurred through the support of unions and government ministerial representatives. The employer and union in each service circulated an email inviting their employees and members to complete the survey designed for their region (i.e., the data set is composed of six surveys for six replicated studies). Due to overlap between listservs in each province/territory, we are unable to estimate sampling frames or determine response rates. The use of both listservs was advantageous in that the combination increased the possibility of sampling the entire population, which we believe outweighed the drawback of this limitation. To be clear, we checked all responses for duplication and found none, thus each respondent is a unique case. The survey could be completed either during paid work hours or in the privacy available outside of work. Respondents could also leave and return to the survey to finish completing it, if desirable or necessary. Participants spent, on average, between 25 and 40 min doing the survey.

### Participants

The survey administered asked respondents at the end of the survey: “If you have any additional information you would like to provide or additional feedback, please feel free to do so below,” which allowed respondents an open field to respond. A total of 1999 correctional workers from Manitoba, Saskatchewan, Nova Scotia, New Brunswick, Newfoundland and Labrador, and Yukon Territory, employed in the community, administration, and institutions, participated in the broader survey, ranging from correctional and probation officers, program officers, nurses, rehabilitative staff, other healthcare staff, administrative staff, managers, teachers, and other employees. Of the 1999 respondents who completed the survey, 192 participants responded to the aforementioned open-ended question with additional feedback. In the qualitative analysis, we only share job category to protect the confidentiality and anonymity of participants. Participants’ identities have been replaced with a unique and confidential numeric identifier, as sharing pseudonyms can problematically imply race, gender, culture, and so forth or overlap with another person in the role. We present select demographics of participants in Table 1.

**Table 1:** Participant demographics.

Sociodemographic information	% (*n*)
Gender	
Man	54.17 (104)
Woman	43.23 (83)
Other/Not specified	2.6 (5)
Occupation group	
Community administrative	0.52 (1)
Institutional administrative	1.56 (3)
Institutional	13.02 (25)
Operational	84.38 (162)
No response	0.52 (1)
Education level	
Highschool or less	19.79 (38)
Less than 4-year postsecondary	32.81 (63)
4-year postsecondary or higher	38.54 (74)
Other/not specified	8.85 (17)
Marital status	
Single	17.71 (34)
(Re-)Married/common law	70.31 (135)
Separated/divorced/widowed	8.85 (17)
Other/not specified	3.13 (6)

### Analysis

Our analytic process was thematically inductive (Hesse-Biber and Leavy, 2003) and employed a constructed semi-grounded emergent theme approach (Charmaz, 2014; Glaser and Strauss, 1967), meaning we did not know what themes would emerge from the data and we framed the study according to what the data revealed theoretically but did not create any theory (Ricciardelli et al., 2010). Using QSR NVivo software, in the first stage, open coding was initiated of the participants’ responses. This process involved research team members independently reading through all responses, breaking down, comparing, and categorizing the data (Strauss and Corbin, 1990). This stage was intended to ensure that the coding process was divorced from any expectations of the results and without influence from previous research interactions with the data. The open coding process was then repeated, as the research team read through the responses a second time with the impressions gained from the first round of open coding.

Once the open coding process was complete, a focused coding process followed by identifying thematic categories, merging similar themes, and finding connections between some themes and categories (Esterberg, 2002). The focus coding stage also allowed the research team to identify lone codes from the open coding stage and not attend to these since they lacked saturation. With thematic categories and connections between them identified, the research team created a description of the themes and noted exemplars from participants’ responses. The data were combined from the items under analysis into one file for ease of analysis to gain a sense of the whole data and the key themes across responses (Corbin and Strauss, 2015).

A theme was operationalized as multiple participants reporting similar interpretations, experiences, or needs—in other words, excerpts from multiple respondents coded in a theme then became an emergent theme. We draw on quotes in the presentation of results that highlight respondent voices but do so ensuring that we neither identify the respondent nor impinge on their privacy. To assist with readability and flow, we did make minor edits to the excerpts used, though we made sure that these did not compromise interpretations, meaning, or tone.

## Results

### Effects of PPTE

When respondents were given the opportunity to set the agenda by sharing information they thought was most pertinent to their mental health and well-being, they often spoke frankly about how the job wore on them. Specifically, respondents described the psychological, physical, and emotional effects of the job. Participant 47 from Manitoba (Correctional Officer), for example, shared that.

In the beginning I knew I would see fights, potentially death, potentially be injured. What I did not expect was that I saw those things, dealt with them, was not seriously bothered by the events. However, years later I feel haunted. I have long since forgotten inmates’ names and faces [who] now come to me in my sleep.

In this excerpt, readers are shown that PPTEs can linger and flare up into psychological harms years later. We also learn of a way that trauma manifests. Names and events once suppressed, return to haunt correctional workers and steal restful sleep. But the PPTE of correctional service work does not just leave a residue that seeps into one’s sleep. This is a psychological trauma that is mentally draining and spills over from the occupational sphere to affect family and personal relationships. To evidence this finding, Participant 367 from Saskatchewan (Correctional Officer) wrote:

Corrections is hard on the body and mind, it can wreck your only support systems through lack of time, energy, and understanding… [I put] myself at risk of physical harm. My mental health normally would act up and down through the year but working corrections has made it act up and stay stuck. It has ruined my marriage, from a decline in mental health but also from not being able to have a partner to speak with openly and earnestly about my job. To be frank, this job sucks.

In total, 18.75% of the responses and 15.25% of the coded themes described the effects of PPTE. Arguably implicit in the above passage, many respondents were striving to create an awareness of mental health needs, seemingly with an intent to increase the recognition of mental health concerns among correctional workers—themes to which we now turn.

### Operational and organizational stigma and the need to better recognize mental health

Some participants (9.89% of the responses and 8.05% of the coded themes) shared that mental health is stigmatized within the profession and the culture encourages correctional workers to suppress attending to one’s mental health. Where stressors within the workplace were often located within organizational factors, respondents locate the stigma around mental health in both operational and organizational elements and describe how stigma is present in correctional work at all levels of governance and administration, including frontline work and management:

Corrections, like Policing, continues to hold a belief that we have to be mentally tough and be able to handle everything we experience. There is still stigma, but at the same time, as staff, we must be mentally strong so as to best serve the people we are in direct care of, be it in custody or in community (Participant 234 from Saskatchewan, Probation Officer).

There are numerous Correctional Officers with mental health issues in our workplace. Some/most are too proud to ask for help. This is fear of what your co-workers will say about you, including management. There are still what is considered ‘old school’ mentality among some managers that would laugh if you said you needed a moment to clear your head. You are met with, ‘What do you need a minute for, this needs to be done…that needs to be done…go to work.’ No amount of training, or mentoring can fix this. The prison runs like clockwork, if it does not, someone pays. And that someone is always at the bottom. Fear mongering would be an ideal way to describe it (Participant 18 from Newfoundland and Labrador, Correctional Officer).

In the first comment, Participant 234 explicitly acknowledges there is stigma, but also illustrates how stigma manifests in their work and in the work of correctional workers in all operational capacities. They believe the best way to serve those under their care is to “be mentally strong.” While this may reify mental health stigma, the correctional worker believes this is necessary to care for those under their supervision, even though mental health needs that are unmet can be a barrier to treatment-seeking and care. Also, the comment reveals operational aspects—“we are in direct care of”—and organizational aspects—the structures within public safety (i.e., correctional services and policing) that normalize the need for mental toughness in the job. There is a need to present as stoic and able to handle diverse situations, which may drive perceptions of a gendered work environment (see Ricciardelli, 2017). In the second comment, Participant 18 shares how many correctional workers will not ask for help because they fear the response asking for help will elicit from coworkers and direct managers. The need for the prison to run “like clockwork,” given it is an institution that runs on necessary routines and structures to maintain institutional safety and order, may encourage stoicism and a need for pushing through PPTE to sustain operational needs.

Other respondents articulated the stigma around mental health with a hope that it might be acknowledged, discussed, and addressed on the job by staff and management. This was best summarized by Participant 224 from Saskatchewan (Probation Officer) who shared:

I think promotion of stable mental health should be advocated more within my Ministry and within the workplace. I believe it is not a topic which is discussed often enough and people tend to be apprehensive when talking about it for fear of how it might look for their position.

If correctional work is no longer to be “a job that honestly takes your soul with it,” as Participant 44 from Manitoba (Probation Officer) stated, then it is necessary to rethink how correctional workers can live and work with their PPTE and related mental health concerns. Participants are clear – there is still a need for change throughout Canadian correctional services with respect to mental health frameworks, which applies to frontline staff and to high-level management.

### Organization factors: vertical relationships

Respondents predominantly from the province of Manitoba identified management as contributing to or acting as a barrier to recovery from their experiences with PPTE. In fact, management was almost two-and-a-half times more likely to be reported as impacting mental health than all other relational aspects combined (i.e., coworkers, those under their supervision, family members, and members of their communities), with 31.77% of the responses and 25.84% of the coded themes identifying relationships with management as causing stress, aggravating PPTE, and acting as a barrier to healing from trauma and other mental health concerns. In our analysis, we included all levels of supervision and oversight, from front line to institutional to government, in our coding of management, which were discussed by some participants in strong terms:

Staff feel VERY STRONGLY that management does not support us in our struggles at work. There is a very REAL perception that we are just “numbers” in a very large system and that if something were to happen to any of us, we would be replaced and forgotten in a SECOND (Participant 236 from Manitoba, Correctional Officer).

Senior management at our corrections facility have been quoted as saying ‘It’s not our problem’ regarding staff suicide AND staff mental health (Participant 217 from Manitoba, Probation Officer).

Participants’ comments here point to feeling unsupported by management. Participants believe their struggles are a result of management’s framing of mental health and suicide as a personal problem. This is not just a framing issue, however, as there is a “perception” by one staff member that an abdication of responsibility toward staff mental health exists within correctional services. When participants believe the onus for mental health rests on individual correctional workers, participants perceive management not only ignores the role of operational factors, but also reinforces structural factors within the organization that fracture its relationship with staff, causing one correctional worker to feel alone in “a very large system.”

Support for mental health arguably includes providing health interventions and a peer support infrastructure. But several participants emphasized that such practices can only be fully realized with strong leadership and people management throughout the organization, beginning at the top. As Participant 37 from Manitoba (Probation Officer) summarized:

We need better management that make strong leaders and lead by example. Recognize staff for doing a good job and actually believe that staff are our greatest resource. Because right now that’s not happening at all. Morale is the lowest I’ve seen at my institution. Upper management is surrounding themselves with managers that tell them what they want to hear but not what’s actually happening at the front line. Major disconnect between management and the frontline. Managers have become more about advancing their careers than to look after their staff. This has resulted in a lot of issues for staff. This culture needs to change in order for the mental health of staff to improve in the long run. This has to come from top down. How many more staff suicides is it going to take for our downtown management to recognize what they are doing is not working and only making matters worse? Please help make Corrections a better place than it is right now.

The current culture is described as resulting in correctional workers feeling the effects of organizational factors. A cultural need for better leadership impacts managers as well. As Participant 186 from Saskatchewan (Supervisor) shared, “most stress for myself is not necessarily the job itself but unreal expectations, lack of communication, respect and recognition or feedback that one receives from those above.”

In addition to poor organizational factors, such as support, communication, and feedback, respondents also described an emerging bullying problem from some managers. While bullying has been identified as a problem within some correctional facilities among peers (Ricciardelli and Carleton, 2022), respondents identified bullying as a problem predominantly within management. Perceived bullying appeared in 5.21% of the responses and 4.24% of the coded themes.

While this is a small percent of responses, the nature of bullying and its effects on mental health makes bullying worthy of discussion. Of the responses that discussed bullying, 80% discussed bullying by managers, and 20% discussed bullying from peers. Participant 419 from Manitoba (Correctional Officer) shares they “work in a place where management bullies people. Where people who say they are having trauma issues are blamed and minimized. I have managers who behave with zero respect toward their staff.” Another respondent, Participant 123 from Manitoba (Correctional Officer), provides insight into how individual managers’ approach to supervision and personal interaction could be difficult and harmful: “The difficult part of the job is the politics with Management and having a Superintendent who refers to them themselves as ‘Little Hitler,’ implying they will adopt whatever method necessary to get Officers to comply.” As discussed previously, there is a need for better mental health support in correctional services more broadly and to address organizational factors that emerge in the social structures and the shifting culture of correctional services. But there is also a need for frontline workers and managers to come together as a team and communicate issues effectively and respectfully to promote a healthy workplace environment.

### Organization factors: time off and sick time

While data reveal a need to address problematic vertical relationships, participants’ responses also made evident that part of the relationship strain resulted from the norms and policies managers enforced. In particular, the policies pertaining to time off, especially sick time, made up a significant portion (28.88%) of the comments coded as health and well-being policy criticisms and suggestions.

This division needs help, lots of it. You need management that are involved not sitting up in a safe desk having no clue what’s actually going on. Try adding bullying to all of this too and have nothing done about any situation that’s ever brought forward. Scenario - say I’m close to being over my sick time (based on an average) have a mental episode or something and I’m now forced to choose between coming into work and or getting in trouble down the line and have my position threatened. Now you have a person feeling forced into work in that mood/crisis? Only a matter of time before something bad happens (Participant 95 from Manitoba, Correctional Officer).

Sick time benefits will always be used and should not be investigated no matter what, even if the Supervisors believe they are being used wrong. What type of manager should be able to dictate what I classify as ‘sick’ and what I do while being away from work. Just be glad that when I am ‘sick’ I do not go to work and do something wrong or criminal (Participant 116 from Saskatchewan, Correctional Officer).

Corrections is not supportive of Mental Health concerns. The Attendance management policy is very anti Mental Health. The whole policy causes added anxiety and stress to any mental health concerns (Participant 438 from Manitoba, Teacher).

These excerpts suggest the ‘time off’ policy is viewed as valuable to respondents because of the role the policy has in adding to correctional workers’ mental health stressors, the interplay between policy and management which are two organizational factors, and the risk the policy adds to the operations of correctional institutions. Above, Participant 116 challenges managers’ discretion to investigate and challenge the use of sick time. The participant believes correctional workers should be granted the chance to discern when they need to take time to rejuvenate and the freedom to take the actions they see as best serving this aim. Participant 95 also focuses on managers, attributing absentee managers as the problem, and the sick time policy, sharing that sick time policy has the potential to lead to something detrimental. But Participant 95’s response is still hopeful and optimistic toward management, suggesting if a manager was involved, the manager would then become aware of the employee’s “mood/crisis” and they could identify the possible harm from this, and send the employee home, overriding the policy of a sick time cap based on average.

### Organization factors: treatment options

Respondents also included criticisms and suggestions pertaining to treatment of mental health for correctional workers, with 16.67% of the responses and 13.56% of the coded themes describing treatment options for correctional worker mental health. We categorized these suggestions into three categories: treatment options, training for peer support, and eliminating institutional monitoring. Correctional workers noted they lacked effective options for dealing with PPTE. Participant 409 from Saskatchewan (Correctional Officer), for example, shares that:

The PTSD treatment that I received for 8 months was not helpful and mostly outdated. Talk therapy does not go far for my trauma related injury. Psycho Dramatic Body Work Therapy was much more useful, and my Psychologist at the CBI Health Clinic had not even heard about it.

While Participant 409’s comment called specifically for psycho-dramatic bodywork therapy, other participants unfolded other possible options that would improve their mental health. Some correctional workers, such as Participant 116 from Manitoba (Correctional Officer), are finding help from services not supported or financed through their employer:

I feel that the department needs access to psychologists who are trained to deal with PTSD and workplace trauma. I have to access free services at the U of M which only last through the student’s current term. After her term is over I am back to where I started which is having no treatment due to not having the funds to pay for a trained psychologist. I understand I can make a claim with WCB, however there is the fear of the claim being rejected and not having the faith or energy to keep fighting.

Not all correctional workers have the same opportunity as Participant 116 to find support through family members’ insurance plans. Further, if correctional workers wish to receive PTSD and PPTE specific support, they may be forced to fund this privately. Participant 116 does mention the possibility of filing a workers’ compensation claim, but there is not a lot of trust that this would be successful and would demand considerable energy to advocate for oneself. Participant 382 from Manitoba (Correctional Officer) also shared that “management should support their workers opposed to conspiring with WCB to save money. It’s bad enough having to deal with the effects of a life changing injury without feeling victimized all over again.”

Regardless of the validity of the claim that managers are working with another government agency to minimize cost, correctional workers feel managers’ scrutiny and oversight of the workers compensation board fuels distrust in the organization. Many respondents suggested treatment access options with fewer barriers to entry. A suggestion from Participant 1 from New Brunswick (Correctional Officer) stated: “Provincial Correctional Officers need to be considered as FIRST RESPONDERS and as such should not have to be assessed or questioned when they require help for PTSD.” Classifying correctional workers as first responders in provinces and territories could provide quicker access to care, which would be beneficial, especially for individuals in crisis. Moreover, recognizing correctional workers for the complexities of their occupational responsibilities and role in public safety may do much to increase morale and qualify correctional workers for immediate care tied to presumptive legislation.

Another suggestion that emerged was, as Participant 15 from Nova Scotia (Administrator) summarized, the “need to have staff with corrections background doing the work with corrections staff as they know the business.” There is merit in having a mental health practitioner with knowledge of correctional work providing support to correctional workers. This could be supplemental to the immediacy of treatment accessed with a first responders’ classification, as well as a peer support person. Manitoba Participant 376’s (Correctional Officer) additional comment demonstrates how peer support can act as an intervention from peers who understand the job and the PPTE:

I am aware that corrections is now starting to acknowledge the mental health damage that is occurring to officers but unfortunately, if they do not utilize a “peer support” angle (people who have actually gone through it therefore “get it”) the approach will never work. COs are a special breed of people and take offence when “program people” try to relate to their experiences because [they] have no idea.

Others suggested training in peer support as informal treatments that are more of a collegial support and enacting a culture that is conducive to better mental health. Participant 115 from Manitoba (Correctional Officer) explains that:

There is a need to have more training around mental health and mental health practises. I feel that we are told about self care but at the same time based on the current political climate we are discouraged from practising self care at work…Again we need to change the mindset of a Culture.

Participant 115’s comment describes cultural shifts that extend into society at large but also include the need for a culture within the correctional workspace that allows for everyone to attend to self-care needs. Other ways that respondents suggested a need for training that would improve collegial peer support was “personal mental health training in recognizing how my co-workers may be affected without them realizing they are being under stress” (Participant 452 from Manitoba, Correctional Officer) and “more training for supervisors on how to deal with when a staff member comes to you with Mental health issues, and a program to reduce stigma (i.e., “that it’s ok to be human and seek help even [though] you are a corrections worker”; Participant 379 from Saskatchewan, Program Staff). In these two suggestions, peer support is not being suggested as therapeutic techniques. Rather, these suggestions point to ways that peers can simply help peers as peers.

## Discussion

Mental health concerns and the presence of mental health disorders are too prevalent among correctional workers in comparison to the general population and often other public safety personnel (Carleton et al., 2018, 2022; Ricciardelli et al., 2018). Respondents described how correctional work wears on them, including psychological, physical, and emotional effects as well as the various ways these effects spill over into their personal and family lives. As other research on correctional services has demonstrated (Johnston et al., 2021, 2022b; Johnston and Ricciardelli, 2023); participants shared how mental health and attending to mental health is still stigmatized within the profession and the need to overcome this stigma to adequately address the mental health concerns that arise from operational and organizational stressors. Here, breaking down barriers to mental health treatment seeking is essential to encourage employees to come forward and seek intervention without fear of occupational or personal repercussions. The correctional workers who answered our open-ended question identified three organizational factors as noteworthy factors: relationships with management, the oversight and implementation of sick time, and existing mental health treatment options available to correctional workers. Thus, if an employee does not feel supported post PPTE by managers and colleagues, there is the possibly for the impacts of the PPTE to be more detrimental on the employee’s mental health and wellness.

Our findings support previous research. The stigma articulated by participants of our study echoes the finding of Johnston et al. (2021) who unpack the ways that correctional workers perform emotional labor (see also Westaby et al., 2020) to conceal their struggles and suffer in silence. Similar to the findings of Ricciardelli et al. (2020), many respondents in our study shared their experience of the stigma around mental health with a hope that sharing might create an awareness on the job by staff and management. Our research also confirms the tensions in many relationships with management (McKendy et al., 2021) and the desire for improved mental health treatment options available to correctional workers (Johnston et al., 2022b), especially the desire to include some form of peer support (Johnston et al., 2021, 2022a; Ricciardelli and Carleton, 2022). To this point, these findings reiterate the need to sensitize managers and leaders in correctional organizations to the importance of mental health and readiness (see Johnston et al., 2023) as well as finding other opportunities (i.e., through technology) to promote mental health awareness, resources, education, treatments, coping skills, work-stress management skills, and so forth to struggling correctional workers (Paginin and Simbula, 2020).

Our study not only supports previous research, generally, but the research also provides evidence of external validity and reliability for the earlier findings—the findings stayed consistent across six regions and different correctional services, which suggests challenges are generalizable and areas worthy of intervention. Recognizing replication is essential to the value of empirical findings, we note how some of the aforementioned research (Ricciardelli et al., 2020; McKendy et al., 2021; Ricciardelli and Carleton, 2022) used data from one Canadian jurisdiction, Ontario, while other research (Johnston et al., 2021, 2022a,b) used data collected from different, more targeted questions from the same jurisdiction as this study. This study, having drawn from the Manitoba, Saskatchewan, Nova Scotia, New Brunswick, Newfoundland and Labrador, and Yukon jurisdictions, confirms the findings from Ontario and shows how these findings are generalizable in Canadian provincial and territorial jurisdictions. Further, because we drew on a single open-ended question that did not solicit any responses about a specific topic and, thereby, afforded respondents the opportunity to voice what was at the fore to them, this study provides evidence of internal validity in the findings of earlier research that drew on targeted questions from the same jurisdictions. Finally, our findings also expand upon the employment, wage, and benefit conditions (Ricciardelli et al., 2020), which included calls to add sick time/leave policies and procedures and clear directives for its effective management and de-stigmatized accessibility.

Respondents discussed how specific operational and organizational factors were detrimental to their mental health (see Ricciardelli and Power, 2020; Norman and Ricciardelli, 2022; Ricciardelli et al., 2023a), which suggests managers, as well as policies and practices, can cause stress, aggravate psychological trauma, and act as a barrier to healing. Such realities are informed by an occupational culture that continues, despite progress that normalize mental health concerns, to stigmatize mental health. The consequence of a less open to mental health culture may encourage correctional workers to suppress, without seeking support or intervention, their own mental health struggles —which may aggravate mental health needs.

Some correctional managers are promoted into their current position – raising through the ranks – thus, these managers are familiar with the operational and organizational aspects of the occupation. The challenge remains that managers are occupationally socialized, learning, and informing a culture that, in many examples but not exclusively, until recently was rather silent about mental health. Thus, current efforts are required to ensure cultural change is from below and above in the correctional hierarchy, encouraging people to speak openly about their health needs and recognize when organizational policies may exacerbate conditions that are detrimental to mental health. Effective change directed toward benefiting the mental health of correctional workers must address the organizational culture, recognizing it is changing but perhaps not quickly enough.

## Conclusion

There is a need to support all correctional workers in recognizing their mental health needs, which is essential to create a space where all correctional workers are open to treatment, intervention, and support. Given the prevalence of mental heath disorders among correctional worker populations, there is also a need to look at oneself and practice self-care as well as address the needs of colleagues. One can only help another if they have the capacity – capacity that may be compromised if one is struggling with their own mental health. Moreover, the culture shift—the openness to discuss mental health—must be signaled to correctional workers and demonstrated through actions and meaningful responses. It is by coming together and recognizing the suffering in the self and other that the silence can be broken, and the healing moved to the forefront of stronger mental health frameworks in correctional services.

## Data availability statement

The datasets presented in this article are not readily available because of reasons surrounding privacy and confidentiality. Requests to access the datasets should be directed to Rose.Ricciardelli@mi.mun.ca.

## Ethics statement

The studies involving humans were approved by research ethics boards at the University of Regina (file #2017-098) and Memorial University of Newfoundland (file #20201330-EX). The studies were conducted in accordance with the local legislation and institutional requirements. The participants provided their written informed consent to participate in this study.

## Author contributions

RC: Conceptualization, Formal analysis, Methodology, Writing – original draft, Writing – review & editing. MJ: Conceptualization, Formal analysis, Methodology, Writing – original draft, Writing – review & editing. RR: Conceptualization, Data curation, Formal analysis, Funding acquisition, Methodology, Project administration, Supervision, Writing – review & editing.
